# Imbalance of Lymphocyte Subsets and CD45RA-Expressing Cells in Intrathoracic Lymph Nodes, Alveolar Compartment and Bloodstream of Pulmonary Sarcoidosis Patients

**DOI:** 10.3390/ijms241210344

**Published:** 2023-06-19

**Authors:** Miriana d’Alessandro, Laura Bergantini, Sara Gangi, Paolo Cameli, Martina Armati, Matteo Fanetti, Fabrizio Mezzasalma, Stefano Baglioni, SARC-SI Study Group, Elena Bargagli

**Affiliations:** 1 Respiratory Diseases Unit, Department of Medical and Surgical Sciences & Neurosciences, University of Siena, 53100 Siena, Italybargagli2@gmail.com (E.B.); 2 Diagnostic and Interventional Bronchoscopy Unit, Cardio-Thoracic and Vascular Department, University Hospital of Siena (Azienda Ospedaliera Universitaria Senese, AOUS), 53100 Siena, Italy; 3 Pneumology Department, Perugia Hospital, 06129 Perugia, Italy

**Keywords:** sarcoidosis, lymphocytes, CD45RA, lung-draining lymph nodes, bronchoalveolar lavage

## Abstract

Sarcoidosis is a systemic granulomatous disease mainly affecting the lungs and hilomediastinal lymph nodes. It is characterized by non-caseating epithelioid cell granulomas in lymph nodes and lungs. Our study aimed to evaluate and compare T, B and NK cell subsets in the alveolar compartment, lymph nodes and the bloodstream simultaneously in the same patients to elucidate the immune responses associated with the development and progression of sarcoidosis. A secondary aim was to evaluate the distribution of CD45RA-expressing cells in the different anatomical compartments. Patients suspected to have sarcoidosis and who underwent bronchoscopy with bronchoalveolar lavage (BAL), lung-draining lymph node (LLN) biopsy by EBUS-TBNA and peripheral blood (PB) sampling were included in the study. They were monitored at the Regional Referral Centre of Siena University Hospital and the Respiratory Diseases Unit of Perugia Hospital. Multicolour flow cytometry analysis through FASCLyric was performed to assess T, B and NK cell subsets. Thirty-two patients (median age (IQR) 57 (52–58) years) were consecutively and prospectively enrolled. Machine learning analysis created a model which selected CD56dim16bright, CD8, Tfc, Th17, Th12, Tfh17, Tfh2, Tc_em_RA, Th_em_RA, T naïve, Tc naïve, Breg, CD1d^+^CD5^+^, Th-reg, Tfh, Th1 and CD4 cells with an accuracy of 0.9500 (kappa 0.8750). Comparative analysis found 18 cell populations that differed significantly between the three anatomical compartments. The bloodstream was enriched in Th_em_RA (*p* = 0.0416), Tfh2 (*p* = 0.0189), Tfh17 (*p* = 0.0257), Th2 (*p* = 0.0212), Th17 (*p* = 0.0177), Th-naïve (*p* = 0.0368), CD56dimCD16bright (*p* < 0.0001), CD8 (*p* = 0.0319), Tc_em_RA (*p* < 0.0001) and Tfc cells (*p* = 0.0004) compared with the alveolar compartment, while Th-reg were lower in PB than BAL (*p* = 0.0329). The alveolar compartment was enriched in Breg (*p* = 0.0249) and CD1d^+^CD5^+^ (*p* = 0.0013) with respect to LLN samples and PB. Conversely, Tfh (*p* = 0.0470), Th1 (*p* = 0.0322), CD4 (*p* = 0.0486) and Tc-naïve (*p* = 0.0009) were more abundant in LLN than in BAL and PB. It has been speculated that changes in the relative contents of PB cells could be related to changes in production and to the selective redistribution of PB cells to granulomatous foci. This study further supports the fact that sarcoidosis is multisystemic in nature. However, the low level of immune cells in peripheral blood of patients with sarcoidosis is concerning. A re-expression of CD45RA on CD4^+^ and CD8^+^ cells could result in a reduction in peripheral immune activity. Thus, changes in the spectrum of the bloodstream may reflect both pathogenic and compensatory processes.

## 1. Introduction

Sarcoidosis is a systemic granulomatous interstitial lung disease (ILD) mainly affecting the lungs and hilomediastinal lymph nodes [1]. It is characterized by non-caseating epithelioid cell granulomas in lymph nodes and lungs [2].

Since the disease has a typical constellation of clinical and chest X-ray findings, a presumptive diagnosis can often be advanced on the basis of a finding of radiological stage I (bilateral hilar and/or mediastinal lymphadenopathy) or stage II (bilateral hilar adenopathy and parenchymal infiltration) associated with specific clinical symptoms [3]. However, in most cases cytohistological confirmation of granulomatous inflammation is still necessary for diagnostic purposes and to exclude malignancy and infection. Sarcoid lung involvement can be evaluated by flexible bronchoscopy with bronchoalveolar lavage (BAL), cytological analysis and evaluation of T cell expression, typically characterized by an increase in alveolar lymphocytes having an elevated CD4/CD8 ratio [4]. For better diagnostic accuracy, it is advisable to perform endobronchial ultrasound-transbronchial needle aspiration (EBUS-TBNA) during flexible bronchoscopy, a minimally invasive technique that allows sampling of lung-draining lymph nodes (LLNs) for cytological analysis [5,6]. This procedure has been widely adopted and is currently recommended by international guidelines as the gold standard for evaluating intrathoracic lymphadenopathy in cases of suspected sarcoidosis [7].

Originally described as a Th1-driven disease, sarcoidosis involves a complex interplay of different immune cells. Cumulative evidence suggests that Th17, regulatory T helper (Th-reg) and follicular T helper (Tfh) cells also play a critical role in the pathogenesis [8,9,10,11]. As a result of the defective protective function of Th-reg cells in patients with autoimmune diseases, tissue tolerance is broken down, and ongoing immune responses do not decrease in a timely manner, as is the case in patients with inflammatory diseases [12].

Evidence that Th17 cells are elevated in the lung and peripheral blood of patients with active sarcoidosis sustains the multisystem nature of the disease but raises the issue of a diminished cell immune response in the blood. Both sarcoidosis and autoimmune diseases showed an activation of innate immunity that led to T reg cell dysfunction and elevated Th-17 cell count in peripheral blood. Indeed, a significant increase in the Th17/Th-reg cell ratio has been observed in BAL and peripheral blood of sarcoidosis patients with active disease [13]. These cell subsets are closely involved in the development of autoimmune diseases. Although sarcoidosis is not considered an autoimmune disease, various triggers have been implicated in its development, including autoantigen-specific T cells, antibodies producing B lymphocytes and autoimmune inflammation [14].

Several triggers including viral infections, inflammation and ageing could induce the accumulation of highly differentiated CD45RA re-expressing memory T cells [15]. There is a decrease in proliferative capacity, an increase in senescence signaling pathways activation, and a greater susceptibility to apoptosis in these cells [16]. No studies reported their role and distribution in sarcoidosis patients.

Previously neglected cells, including B lymphocytes and natural killer (NK) cells, have recently been studied to clarify their contribution to sarcoid granuloma formation [4,17]. Imbalance in B cell subsets could be linked to the abnormal distribution of Tfh cells, known to have distinct capacities to help B cells [18]. When NK and T cell subsets were recently characterized, sarcoidosis patients showed a distinct phenotype of these subsets in the bloodstream and lungs [19].

To our knowledge, a detailed comparison of their simultaneous distribution in three anatomical compartments is still lacking. Data on the distribution of T, B and NK lymphocyte subpopulations could help clarify immune response activity by defining the way certain functional cell populations are recruited to the site of damage.

Our study aimed to evaluate and compare T, B and NK cell subsets in BAL, LLN and peripheral blood (PB) simultaneously in the same patients in order to elucidate aspects of the immune responses associated with the development and progression of sarcoidosis. A secondary aim was to evaluate the distribution of CD45RA-expressing cells in the different anatomical compartments.

## 2. Results

### 2.1. Study Population

Thirty-two patients (median age (IQR), 57 (52–58) years) were consecutively and prospectively enrolled in the study. Clinical and immunological findings are reported in Table 1.

Nineteen patients (59%) were females and 21 (66%) were never-smokers. At onset, 12 patients (37%) reported clinical symptoms including cough, dyspnoea, chest pain and joint pain. All patients were in Scadding radiological stage II with enlargement of lymph nodes and interstitial lung abnormalities. None of them were taking oral steroids or inhaled therapy at the time of sampling. BAL cell pattern showed lymphocytosis with elevated CD4/CD8 ratios.

### 2.2. Multivariate Analysis

**Figure 1:**
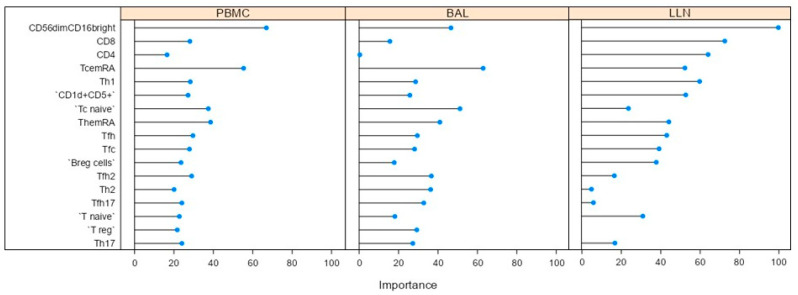
Variable importance plot obtained by machine learning analysis indicates how much a given model “uses” that variable to make accurate predictions. The model selected CD56dim16bright, CD8, Tfc, Th17, Th12, Tfh17, Tfh2, Tc_em_RA, Th_em_RA, T naïve, Tc naïve, Breg, CD1d^+^CD5^+^, Th-reg, Tfh, Th1 and CD4 cells with an accuracy of 0.9500 (kappa 0.8750).

Machine learning analysis with variable importance plot was used to select variables: it indicates how much a given model “uses” that variable to make accurate predictions. The more a model relies on a variable to make predictions, the more important it is for the model. The model (Figure 1) selected CD56dim16bright, CD8, Tfc, Th17, Th12, Tfh17, Tfh2, Tc_em_RA, Th_em_RA, T naïve, Tc naïve, Breg, CD1d^+^CD5^+^, Th-reg, Tfh, Th1 and CD4 cells with an accuracy of 0.9500 (kappa 0.8750).

**Figure 2:**
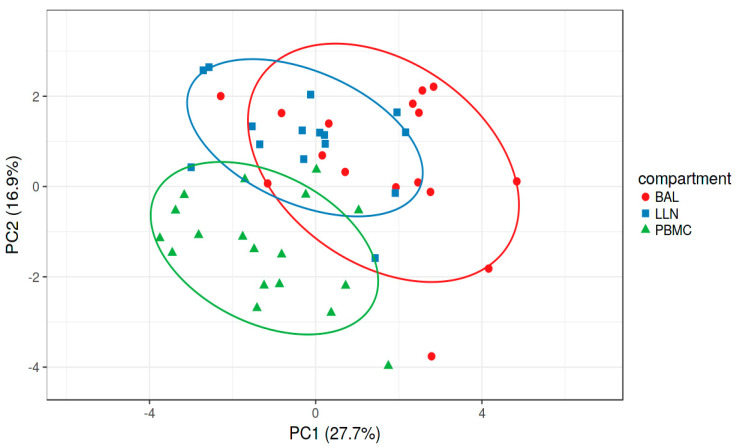
Supervised Principal Component Analysis plot using CD56dim16bright, CD8, Tfc, Th17, Th12, Tfh17, Tfh2, Tc_em_RA, Th_em_RA, T naïve, Tc naïve, Breg, CD1d^+^CD5^+^, Th-reg, Tfh, Th1 and CD4 cells to distinguish the three anatomical compartment: lung-draining lymph nodes (LLN), bronchoalveolar lavage (BAL) and peripheral blood (PB) with total variance of 44.6%.

The supervised Principal Component Analysis plot (Figure 2) shows how the three groups (PB, LLN, BAL) separated on the basis of selected variables. The first and second components explained 44.6% of the total variance.

### 2.3. Comparative Analysis

Comparative analysis found 18 cell populations that differed significantly between the three anatomical compartments. Cell proportions of Th_em_RA (*p* = 0.0416), Tfh2 (*p* = 0.0189), Tfh17 (*p* = 0.0257), Th2 (*p* = 0.212), Th17 (*p* = 0.0177) and Th-naïve (*p* = 0.0368) were higher in PB than BAL samples, while Th-reg were lower in PB than BAL (*p* = 0.0329). We observed significantly higher proportions of CD56dimCD16bright (*p* < 0.0001), CD8 (*p* = 0.0319), Tc_em_RA (*p* < 0.0001) and Tfc cells (*p* = 0.0004) in PB than in BAL and LLN samples.

The alveolar compartment was enriched in Breg (*p* = 0.0249) and CD1d^+^CD5^+^ (*p* = 0.0013) with respect to LLN samples and PB. Conversely, Tfh (*p* = 0.0470), Th1 (*p* = 0.0322), CD4 (*p* = 0.0486) and Tc-naïve (*p* = 0.0009) were more abundant in LLN than in BAL and PB. NK-, Tc- and Tfc-subsets were detected in few LLN samples and were not included in the statistical analysis comparing the three compartments.

## 3. Discussion

Here we analysed the percentages of T-, B- and NK-cell subsets in three anatomical compartments of sarcoidosis patients: alveolar, lymph nodes and peripheral blood. The liquid-based cytology test in intrathoracic lymph nodes sampled by EBUS-TBNA was performed by flow cytometry, identifying the cell subsets involved in granuloma formation.

Machine learning analysis allowed us to construct a model that selected CD56^dim^16^bright^, CD8, Tfc, Th17, Th12, Tfh17, Tfh2, Tc_em_RA, Th_em_RA, T naïve, Tc naïve, Breg, CD1d^+^CD5^+^, Tfc17.1, Th-reg Tfh, Th1 and CD4 cells to make accurate predictions and variables that could be dropped from the model because they did not contribute much information. Multivariate analysis of the data reduced the number of dimensions and a total variance of 44.6% was obtained based on T, B and NK cell subsets showing good clustering variables for LLNs, BAL and PB.

Our current knowledge of the pathogenesis of sarcoidosis includes sequential immunological events triggering activation of T and B lymphocytes. This eventually leads to their migration to inflammatory foci and the formation of granulomas [20]. The present study confirmed data in the literature on depletion of CD56^dim^16^bright^ and CD8^+^ T cells in LLN and BAL samples with respect to PB, suggesting that these cells play a protective role in sarcoidosis [5,17].

In line with previous reports, our cohort showed significantly lower percentages of CD4^+^ T cells in PB than in LLN, further substantiating the crucial role of Th-cell interaction with antigen-presenting cells for granuloma formation and maintenance [6]. Moreover, our study is the first to describe altered Th-CD45RA^−^ subsets in three anatomical compartments of sarcoidosis patients with higher Th2 and Th17 cell percentages in PB than in BAL samples, and higher percentages of Th1 and Treg in LLN and BAL samples than in PB, respectively. Besides supporting the multisystem nature of the disease [21,22], these findings raise concerns about low cell immunity in peripheral blood of sarcoidosis patients [23]. The regulatory counterpart of Th cells accumulating in the alveolar compartment may be due to peripheral recruitment; nevertheless, the proportion of regulatory CD127-expressing cells (Th naïve) was lower in the alveolar compartment than in PB, probably because pathogenic T cells are resistant to suppression signalling by Treg cells [24]. Assessment of CD127 expression on CD8-positive cells (Tc naïve) showed that Tc naïve cell percentages were lower in BAL than in PB and LLN.

Concerning B cells, our study is the first to report a higher percentage of CD5-expressing cells in BAL than in PB and LLN samples. However, the role of this finding remains unclear in sarcoidosis. In many autoimmune disorders, CD5^+^ B cells are reported to expand and to be a major source of natural polyreactive and autoreactive autoantibodies [25]. CD5^+^ B cells must therefore contain a wealth of information about the autoimmune system that has yet to be discovered and analysed [26]. The development of therapeutic strategies to downregulate CD5^+^ B cells so as to mitigate ongoing autoimmunity and protect high risk individuals could be achieved if it turns out that autoimmune repertoires are concentrated in CD5^+^ B cells, the latter being readily identified by flow cytometry.

An imbalance in B cells may be related to the abnormal distribution of Tfh cells, found in secondary lymphoid organs, including lymph nodes, and known to have different functions, including producing plasma and memory B cells through germinal centre reactions [8,18]. Interestingly, it has been reported that Tfh cells infiltrate sarcoid skin lesions, suggesting that they are recruited to affected tissues [27]. In line with this, our study found higher percentages of Tfh cells in LLN samples than in PB and BAL of sarcoidosis patients. An abnormal distribution of Tfh-CD45RA^−^ subsets has been linked to the pathogenesis of sarcoidosis [8,27]. Although all the lymphocyte populations mentioned above are functional cells, as far as non-expression of CD45RA is concerned, a portion of cells re-expresses CD45RA. In our study, the percentages of Th_em_RA and Tc_em_RA cells were higher in PB than in BAL or LLN. Re-expression of CD45RA on CD4^+^ and CD8^+^ cells could mean lower immune activity at peripheral level [28,29] and higher activity in the organs targeted by the disease (alveolar and lymph node compartments). Thus, inflammation is localised at the site of damage, which recruits cells from the peripheral blood where they decrease in favour of the target organ.

The present study is the first to compare the distribution of ‘regulatory’ Tfh1 and ‘pro-inflammatory’ Tfh2 and Tfh17 subsets in three anatomical compartments at the same time from sarcoidosis patients. Like Th2 and Th17, higher percentages of Tfh2 and Tfh17 cells were recorded in PB than in BAL samples. A hallmark of systemic autoimmune disease, as well of sarcoidosis, is the predominance of the Tfh17 subset over Tfh1 cells [9,30]. These findings suggest that targeting Tfh cells could be a therapeutic strategy for sarcoidosis, like the highly effective blockade of the main ligand for CXCR5 receptors in experimental models of rheumatoid arthritis and multiple sclerosis.

The present results improve our understanding of the immunological pathways involved in the pathogenesis of sarcoidosis and warrant a larger cohort study. One limit of our study was that healthy controls could not be included for ethical reasons.

There has been speculation that changes in the relative content of peripheral blood cells may be due to changes in cell production and to selective redistribution to granulomatous foci. This implies that any changes to the spectrum of peripheral blood cells may reflect both pathogenic and systemic compensatory processes.

## 4. Materials and Methods

### 4.1. Study Population

Patients suspected to have sarcoidosis and monitored at the Regional Referral Centre of Siena University Hospital and Respiratory Diseases Unit of Perugia Hospital were consecutively recruited for the study. All patients underwent bronchoscopy with BAL, lung-draining lymph node (LLN) biopsy by EBUS-TBNA and peripheral blood sampling. Diagnosis of sarcoidosis was made by multidisciplinary evaluation of clinical features, radiological features and non-caseating granulomas in lymph nodes and/or endobronchial biopsy specimens according to international ATS/ERS/WASOG criteria [31,32]. Patients with lymph node calcifications, or whose PB, BAL and LLN samples were not collected simultaneously, were excluded from the study. Prior to bronchoscopy and biopsy (by EBUS-TBNA reported in Supplementary Materials Table S1), high resolution computed tomography of the chest revealed lymph node enlargement in all patients included in the study.

Written informed consent to participate in the study was obtained from all patients. Healthy controls were not enrolled for ethical reasons. The study was approved by the regional ethics committee of Siena, Italy (C.E.A.V.S.E. Markerlung 17431 approved on 15 June 2020).

### 4.2. Single Cell Preparations from Blood, Bronchoalveolar Lavage Fluid and Lymph Nodes

Peripheral blood mononuclear cells (PBMCs) were isolated from blood collected in EDTA tubes according to the manufacturer’s protocol [33]. BAL samples were kept on ice, filtered through a 100 μm nylon filter (Syntab) and centrifuged at 400× *g* for 15 min. LLN aspirates were filtered through a 40 μm nylon cell strainer and centrifuged at 300× *g* for 10 min. Lysis of red blood was performed with 1× BD FACS lysing solution (BD Biosciences, San Jose, CA, USA) for 5 min; the cells were then centrifuged at 300× *g* for 5 min. Cells were counted manually and Trypan Blue was used to assess viability as previously reported. All samples showed at least 95% cell viability.

### 4.3. Flow Cytometry Gating Strategy

Multicolour flow cytometry analysis was performed to assess T, B and NK cell subsets using the mAbs reported in Table 2. The cell subsets were characterized by FASCLyric (BD Biosciences, CA, USA) and they were summarized in Table S1.

**Figure 3:**
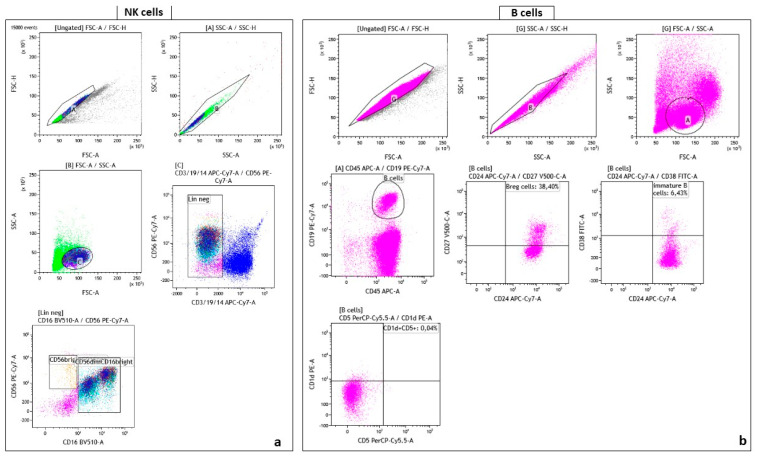
(**a**) The gating strategy of NK cell subsets distinguishing CD56^bright^CD16^dim^ and CD56^dim^CD16^bright^; (**b**) The gating strategy of B cell subsets distinguishing regulatory B cells (Breg), immature B cells and double-positive CD1d and CD5 cells.

The gating strategy performed by Kaluza Software 2.1 (Beckman Coulter, Brea, CA, USA). For the analysis of cells, the total NK cell population was identified based on FSC vs SSC and negative for CD3, CD14, and CD19. CD56 was plotted against CD16 to obtain immature (CD56^bright^CD16^neg^) and mature (CD56^dim/neg^CD16^bright^) phenotypes of NK cells (Figure 3a).

The B cells was identified based on population that express CD45^+^ APC/CD19^+^ PECy7. To obtain B regulatory cells CD27 was plotted against CD24, while the population CD38^+^/CD24^+^ was identify as immature B cells. Regulatory B cells was identified as CD1^+^/CD5^+^ (Figure 3b).

T tubes was assessed for lymphocytes discriminated on the basis of the forward (FCS) versus side (SSC) scatter. A dot plot was performed to distinguish CD8 from CD4 expressing cells. Using CD45RA as marker was identify two CD4 subpopulation CD4^+^45RA^−^ (Th_cm_) and CD4^+^45RA^+^ (Th_em_). From CD4^+^45RA^−^ that expressed CCR6 were identify the T cells helper 17 (CCR4^+^CXCR3^−^) and T cells helper 17.1 (CCR4^−^CXCR3^+^). A dot plot was performed for CD4^+^45RA^−^CCR6^−^ to discriminate T cells helper 2 (CCR4^+^CXCR3^−^) and T cells helper 1 (CCR4^−^CXCR3^+^). In another dot plot the CD4 expressing CXCR5 was identify as T follicular helper cells, then discriminated the Tfh CD45RA^−^CCR6^+^ were distinguished the CCR4^+^CXCR3^−^ (T follicular helper 17 cells) and CCR4^−^CXCR3^+^ (T follicular helper 17.1 cells). Otherwise from the Tfh CD45RA^−^CCR6^−^ were discriminate the T follicular helper 2 cells (CCR4^+^CXCR3^−^) and the T follicular helper 1 cells (CCR4^−^ CXCR3^+^).

Using CD45RA marker was assessed a dot plot on CD8-positive cells identifying CD8^+^45RA^−^ (Tc_cm_) and CD8^+^45RA^+^ (Tc_em_).

**Figure 4:**
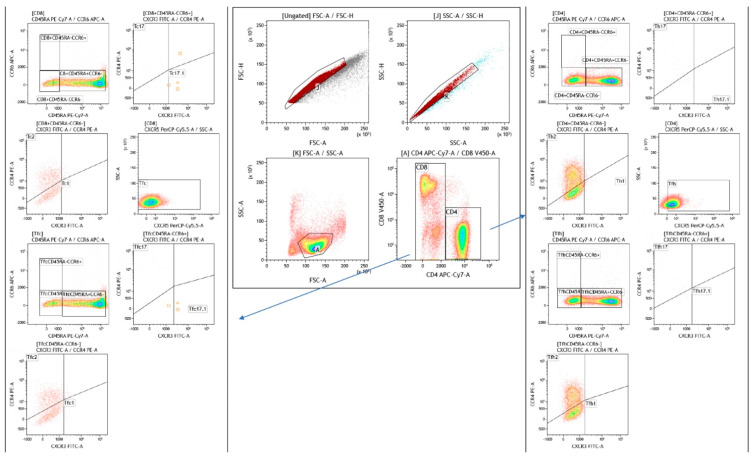
The gating strategy of follicular and CD45-expressing cells distinguishing follicular CD4 cells (Tfh) and the three subsets: Tfh1, Tfh2, Tfh17, Tfh17.1. Additionally, follicular CD8 cells (Tfc) and the three subsets: Tfc1, Tfc2, Tfc17, Tfc17.1.

From CD8^+^CD45RA^−^CCR6^+^ the T cells cytotoxic 17 (CCR4^+^CXCR3^−^) and the T cells cytotoxic 17.1 (CCR4^−^CXCR3^+^) were discriminated. From CD8^+^45RA^−^CCR6^−^ the population that expressed CCR4^+^CXCR3^−^ was identify as T cells cytotoxic 2 and T cells cytotoxic 1 (CCR4^−^CXCR3^+^). A dot plot was performed to identify CD8 cells expressed CXCR5 (T follicular cytotoxic), from that was selected the Tfc CD45RA^−^ population distinguished two sub-population, the Tfc CD45RA^−^CCR6^−^ and the the Tfc CD45RA^−^CCR6^+^. From the last one population the cells that express CCR4^+^CXCR3^−^ was classify as T follicular cytotoxic 17 cells and CCR4^−^CXCR3^+^ (T follicular cytotoxic 17.1 cells). While from the Tfc CD45RA^−^CCR6^−^ was discerned the T follicular cytotoxic 2(CCR4^+^CXCR3^−^) and T follicular cytotoxic 1 (CCR4^−^CXCR3^+^) (Figure 4).

**Figure 5:**
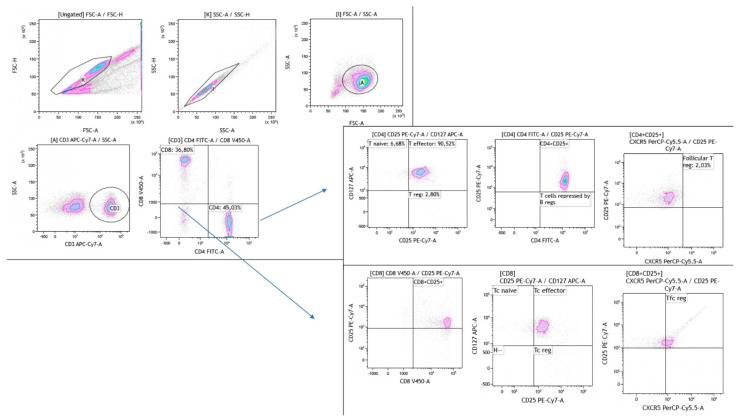
The gating strategy of regulatory CD4 (Threg) cell subsets including T naïve and T effector. Additionally, regulatory CD8 (Tcreg) cell subsets including Tc naïve and Tc effector.

Using CD25 and CD127 markers, a dot plot was assessed on CD4-positive cells to discriminate three different subtypes of T cells: T helper regulatory (Th-reg, CD4^+^CD25^+^CD127^−^), T helper effector (CD4^+^CD25^+^CD127^+^) and T helper naïve (CD4^+^CD25^−^CD127^+^). The gating strategy also included another dot plot which identified a Th-reg population. Three different subsets of CD8-positive cells were distinguished according to CD25 and CD127 markers: T cytotoxic regulatory (Tc-reg, CD8^+^CD25^+^CD127^−^), T cytotoxic effector (CD8^+^CD25^+^CD127^+^) and T cytotoxic naïve (CD8^+^CD25^−^CD127^+^) (Figure 5).

### 4.4. Statistical Analysis

All data is reported as median and interquartile range (IQR) or mean ± standard deviation, as appropriate. The Shapiro-Wilk test was used to determine normal distribution. Multiple comparisons were assessed by non-parametric one-way ANOVA (Kruskal-Wallis test) and the Dunn test. Correlations between immunological and clinical findings were investigated by Spearman’s test. Machine learning and variable importance analysis were performed to construct a model to select variables for accurate predictions based on the cell subsets identified in BAL, LLN and PB. Supervised Principal Component Analysis was performed in an explorative approach to identify trends in immunological features by 2D representation of the multi-dimensional data set. A *p*-value less than 0.05 was considered statistically significant. Statistical analysis was performed with GraphPad Prism 9.3 and Jamovi software 2.3.

## Figures and Tables

**Table 1:** Demographic, clinical and immunological features of sarcoidosis patients enrolled in the study, including age, gender, scadding stage and BAL cellular patterns. Data were expressed as mean ± standard deviation.

Parameters	Sarcoidosis Patients n = 32
Gender (F/M)	19/13
Median age (IQR), years	57 (52–58)
Smoke (never/smoker)	21/11
Radiological Scadding stage II	32/32
BAL cellular pattern:	
Macrophages	72 ± 12
Lymphocytes	19 ± 4
Neutrophils	4 ± 1
Eosinophils	1.5 ± 1.3
CD4/CD8 ratio	4.21 ± 2

**Table 2:** Monoclonal antibody features, including clone, isotype, fluorochrome and company name used for the analysis of NK-, B-, T follicular and T regulatory cell subsets.

CD	Alternative Name	Clone	Isotype	Fluorochrome	Company
CD3	T3, CD3ε	UCHT1	IgG1	APC-Cy7	Biolegend (San Diego, CA, USA)
CD14	Leu-M3, LPS-R	HCD14	IgG1	APC-Cy7	Biolegend
CD16	FCRγIII	3G8	IgG1	BV510	Biolegend
CD19	B4	HIB19	IgG1	APC-Cy7	Biolegend
7-AAD				PerCPCy5	Miltenyi (Bergisch Gladbach, Germay)
CD56	NCAM	HCD56	IgG1	PE-Cy7	Biolegend
CXCR3	CXCR3, CD183, CKR-L2, CMKAR3, GPR9, IP10-R, Mig-R, MigR	REA 232	IgG1	FITC	Miltenyi
CCR4	CKR4, K5-5, CMKBR4, ChemR13, CC-CKR-4, MGC88293, HGCN:14099	L291H4	Mouse IgG1, κ	PE	BD (Franklin Lakes, NJ, USA)
CD45RA				PECy7	
CCR6	CCR6, BN-1, CKRL3, CMKBR6, DCR2, DRY6, GPR29, GPRCY4, STRL22	REA 190	IgG1	APC	Miltenyi
CD4	T4, Leu-3, CD4mut	REA 623	IgG1	APC-Cy7	Miltenyi
CD8	Leu-2, T8	REA734	IgG1	VioBlue	Miltenyi
CXCR5		J252D4		PerCPCy5	Biolegend
Treg-cocktail (CD4/25/127)				FITC/PE/PECy7	BD
CD38	T10; ADP-ribosyl cyclase 1; Cyclic ADP-ribose hydrolase 1; OKT10	HB-7	Mouse IgG1, κ	FITC	BD
CD1d	R3G1	SK9	Mouse IgG2b, κ	PE	BD
CD19	B4, Leu-12	REA 675	IgG1	PECy7	Miltenyi
CD45	Ptprc, GP180, L-CA, LY5, T200	REA 747	IgG1	APC	Miltenyi
CD24	Heat Stable Antigen Homologue (HSA); Ba-1; CD24A	ML5	Mouse IgG2a, κ	APC-Cy7	BD
CD27	S152, T14, TNFRSF7	M-T271	Mouse IgG1, κ	BV510	Biolegend
CD5	LEU1; Leu-1; Lymphocyte antigen T1; T1; LY1; Tp67	L17F12	Mouse BALB/c IgG2a, κ	PerCPCy5	BD

## Data Availability

The data presented in this study are available upon request from the corresponding author.

## Supplementary Materials

The following supporting information can be downloaded at: https://www.mdpi.com/article/10.3390/ijms241210344/s1.

## Abbreviations

ILD: interstitial lung disease; CD-, cluster of differentiation; EBUS-TBNA, endobronchial ultrasound-transbronchial needle aspiration; LLN, lung-draining lymph nodes; Th-, T helper; Tc-, T cytotoxic; Tfh-, T follicular helper; Tfc-, T follicular cytotoxic; Th-reg, T helper regulatory; *p* value, probability value; BAL, bronchoalveolar lavage; PB, peripheral blood.
