# Supplementary material for: Imbalance of Lymphocyte Subsets and CD45RA-Expressing Cells in Intrathoracic Lymph Nodes, Alveolar Compartment and Bloodstream of Pulmonary Sarcoidosis Patients
Source: Int J Mol Sci. 2023 Jun 19;24(12):10344. doi: 10.3390/ijms241210344 (PMC10299444; doi:10.3390/ijms241210344)
Supplement: Supplementary file 1 [file ijms-24-10344-s001.zip › ijms-2434640-supplementary.pdf]

EndoBronchial UltraSound-guided TransBronchial Needle Aspiration (EBUS-TBNA)

Venous blood (32 ml) was drawn from each patient into anticoagulant EDTA tubes (BD Vacutainer®, BD Biosciences, CA, USA) before they were treated with Fentanyl 100 mcg and midazolam (3-5 mg) i.v. 15-30 min prior to bronchoscopy. Lidocaine was administered to the larynx and bronchi for topical anaesthesia. A Pentax bronchoscope EB15-J10 (Pentax Medical Company, PENTAX Europe GmbH, Hamburg Germany) was inserted through the mouth to avoid blood contamination. BAL with 3 × 50 ml saline solution was instilled in the middle or lingual lobe. EBUS-TBNA using linear EBUS EB-1970UK (Pentax Medical Company, PENTAX Europe GmbH, Hamburg Germany) was then performed to sample mediastinal and hilar LLNs. LLN aspirate was obtained using Echotip Ultra (ECHO-HD-22-EBUS, Cook Medical, Ireland). A single-pass sample (5 ml) was obtained for laboratory analysis.

Table S1. The cell subsets characterized by FASCLyric (BD Biosciences, CA, USA) and identified according to surface markers. Abbreviations: CD-, cluster of differentiation; NK-, natural killer.

**Surface markers**	**Cell subsets**
$CD56^{bright}CD16^{neg}$	Immature NK
$CD56^{dim/neg}CD16^{bright}$	Mature NK
$CD19^{+}$	B cells
$CD19^{+}CD24^{+}CD27^{+}$	Regulatory B cells
$CD19^{+}CD24^{+}CD38^{+}$	Immature B cells
$CD19^{+}CD5^{+}CD1d^{+}$	Regulatory B $CD1d^{+}CD5^{+}$
$CD4^{+}$	T helper (Th)
$CD4^{+}CD45RA^{-}$	Th $_{central\ memory}$ (Th_{cm})
$CD4^{+}CD45RA^{+}$	Th $_{effector\ memory}$ ($Th_{em}RA$)
$CD4^{+}CD45RA^{-}CCR6^{+}CCR4^{+}CXCR3^{-}$	Th17
$CD4^{+}CD45RA^{-}CCR6^{+}CCR4^{-}CXCR3^{+}$	Th17.1
$CD4^{+}CD45RA^{-}CCR6^{-}CCR4^{+}CXCR3^{-}$	Th2
$CD4^{+}CD45RA^{-}CCR6^{-}CCR4^{-}CXCR3^{+}$	Th1
$CD4^{+}CXCR5^{+}$	T follicular helper (Tfh)
$CD4^{+}CXCR5^{+}CD45RA^{-}CCR6^{+}CCR4^{+}CXCR3^{-}$	Tfh17
$CD4^{+}CXCR5^{+}CD45RA^{-}CCR6^{+}CCR4^{-}CXCR3^{+}$	Tfh17.1
$CD4^{+}CXCR5^{+}CD45RA^{-}CCR6^{-}CCR4^{+}CXCR3^{-}$	Tfh2
$CD4^{+}CXCR5^{+}CD45RA^{-}CCR6^{-}CCR4^{-}CXCR3^{+}$	Tfh1
$CD4^{+}CD25^{+}CD127^{-}$	Th regulatory (Th-reg)
$CD4^{+}CD25^{+}CD127^{+}$	Th effector
$CD4^{+}CD25^{-}CD127^{+}$	Th naïve
$CD4^{+}CD25^{+}CD127^{-}CXCR5^{+}$	Tfh-reg
$CD8^{+}$	T cytotoxic (Tc)
$CD8^{+}CD45RA^{-}$	Tc $_{central\ memory}$ (Tc_{cm})
$CD8^{+}CD45RA^{+}$	Tc $_{effector\ memory}$ ($Tc_{em}RA$)

$CD8^+CD45RA^-CCR6^+CCR4^+CXCR3^-$	Tc17
$CD8^+CD45RA^-CCR6^+CCR4^-CXCR3^+$	Tc17.1
$CD8^+CD45RA^-CCR6^-CCR4^+CXCR3^-$	Tc2
$CD8^+CD45RA^-CCR6^-CCR4^-CXCR3^+$	Tc1
$CD8^+CXCR5^+$	T follicular cytotoxic (Tfc)
$CD8^+CXCR5^+CD45RA^-CCR6^+CCR4^+CXCR3^-$	Tfc17
$CD8^+CXCR5^+CD45RA^-CCR6^+CCR4^-CXCR3^+$	Tfc17.1
$CD8^+CXCR5^+CD45RA^-CCR6^-CCR4^+CXCR3^-$	Tfc2
$CD8^+CXCR5^+CD45RA^-CCR6^-CCR4^-CXCR3^+$	Tfc1
$CD8^+CD25^+CD127^-$	Tc regulatory (Tc-reg)
$CD8^+CD25^+CD127^+$	Tc effector
$CD8^+CD25^-CD127^+$	Tc naïve
$CD8^+CD25^+CD127^-CXCR5^+$	Tfc-reg
